# Factors associated with human West Nile virus infection in Ontario: a generalized linear mixed modelling approach

**DOI:** 10.1186/s12879-018-3052-6

**Published:** 2018-03-27

**Authors:** Shruti Mallya, Beate Sander, Marie-Hélène Roy-Gagnon, Monica Taljaard, Ann Jolly, Manisha A. Kulkarni

**Affiliations:** 1 School of Epidemiology & Public Health, University of Ottawa, 600 Peter Morand Cres, Ottawa, ON Canada; 2 Institute of Health Policy, Management and Evaluation, University of Toronto, Toronto, ON Canada; 3 Public Health Ontario, Toronto, ON Canada; 4 Institute for Clinical and Evaluative Sciences, Toronto, ON Canada; 5 Clinical Epidemiology Program, Ottawa Hospital Research Institute, Ottawa, ON Canada

**Keywords:** West Nile virus, Climate, Epidemiology, Public health, Risk, Model

## Abstract

**Background:**

West Nile Virus (WNV) is a mosquito-borne pathogen that has become established in North America. Risk for human infection varies geographically in accordance with climate and population factors. Though often asymptomatic, human WNV infection can cause febrile illness or, rarely, neurologic disease. WNV has become a public health concern in Canada since its introduction in 2001.

**Methods:**

To identify predictors of human WNV incidence at the public health unit (PHU) level in Ontario, Canada, we combined data on environmental and population characteristics of PHUs with historical mosquito and human surveillance records from 2002 to 2013. We examined the associations between annual WNV incidence and monthly climate indices (e.g. minimum and maximum temperature, average precipitation), land cover (e.g. deciduous forest, water), population structure (e.g. age and sex composition) and the annual percentage of WNV-positive mosquito pools from 2002 to 2013. We then developed a generalized linear mixed model with a Poisson distribution adjusting for spatial autocorrelation and repeat measures. Further to this, to examine potential ‘early season’ predictors of WNV incidence in a given year, we developed a model based on winter and spring monthly climate indices.

**Results:**

Several climate indices, including mean minimum temperature (^o^ C) in February (RR = 1.58, CI: [1.42, 1.75]), and the annual percentage of WNV-positive mosquito pools (RR = 1.07, CI: [1.04, 1.11]) were significantly associated with human WNV incidence at the PHU level. Higher winter minimum temperatures were also strongly associated with annual WNV incidence in the ‘early season’ model (e.g. February minimum temperature (RR = 1.91, CI: [1.73, 2.12]).

**Conclusions:**

Our study demonstrates that early season temperature and precipitation indices, in addition to the percentage of WNV-positive mosquito pools in a given area, may assist in predicting the likelihood of a more severe human WNV season in southern regions of Ontario, where WNV epidemics occur sporadically.

**Electronic supplementary material:**

The online version of this article (10.1186/s12879-018-3052-6) contains supplementary material, which is available to authorized users.

## Background

West Nile virus (WNV) is a global emerging infectious disease. Initially characterized in Uganda in 1937, WNV first appeared in North America in 1999 in New York, USA, with subsequent spread to Canada in 2001 [1]. The lifecycle of this flavivirus is sustained in a mosquito-avian enzootic cycle, with spillover to humans and other mammals [2]. The majority of human infections are asymptomatic, although approximately 20% of infections cause febrile illness and 1% of infections lead to neuroinvasive disease [1, 3].

In Ontario, Canada, WNV incidence peaks in May to October each year corresponding to the period of mosquito and virus activity. High incidence years occur infrequently, with epidemics in 2002 and 2012 resulting in high WNV incidence rates, of 3.5 and 2.0 WNV cases per 100,000 population, respectively [4]. Importantly, the sporadic nature of the disease poses a challenge for planning and sustaining public health surveillance and intervention strategies to prevent human infection.

Environmental factors, such as climate and land cover, have an important influence on WNV transmission through their effects on mosquito population dynamics and ecology. Higher winter temperatures often correspond with increased mosquito abundance, mosquito biting rates, viral replication and rates of transmission [5–9], while grasslands, wetlands and urban cover have all been associated with WNV activity [10–12]. Notably, the relative importance of different environmental factors can vary greatly across large geographic areas due to differences in primary vector species. In western Canada, *Culex tarsalis* is the predominant vector species, while in eastern Canada, *Culex pipiens/restuans* dominates [13]. These two species have different lifecycles and habitat preferences, which in turn affect the nature of environmental risk factors for disease transmission.

In addition to the environment, individual and population characteristics, such as age, sex, and behavioural risk factors can also influence the incidence of WNV in a given area. Human behaviour, and hence contact with mosquitoes, can vary greatly within and between population groups [14]. Variance in population structure between areas, such as the male/female ratio of a region [15] and the number of senior households in a given area [10] have been identified as predictors of WNV incidence. Importantly, the spatial dependence of factors that contribute to WNV incidence requires consideration of geographically-specific risk factors.

In Ontario, a limited number of studies have investigated local predictors of high WNV incidence seasons, i.e. years with elevated numbers of reported WNV cases during the May to October transmission season. This study aimed to determine PHU-level predictors of annual WNV incidence specific to Ontario, recognizing that early identification of WNV risk can assist PHUs in tailoring their surveillance and response efforts. Herein we used a mixed modelling approach combining climate, land cover, population and surveillance data to ascertain environmental and population predictors of human WNV incidence in southern Ontario.

## Methods

### Data sources

#### Epidemiological data

**Fig. 1:**
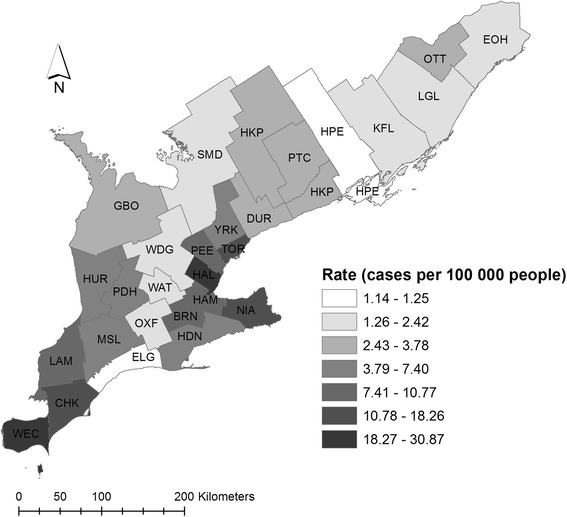
Map of cumulative human WNV incidence (cases per 100,000) in southern Ontario Public Health Units (PHUs), 2002–2013

We obtained data on confirmed and probable human WNV cases in Ontario from 2002 to 2014 from the Integrated Public Health Information System (iPHIS) of Public Health Ontario (PHO), which documents cases of reportable diseases [16]. Cases are reported to the medical officer of health by laboratories and physicians and these data are updated in iPHIS on a weekly basis. Due to low incidence rates, we aggregated weekly case counts to obtain yearly case numbers for each PHU. We restricted our analysis to the 29 PHUs in the southern portion of Ontario (Fig. 1), since northern PHUs have low climatic suitability for the virus and vector and hence few cases of WNV.

#### Population data

We obtained 2011 census populations for each PHU from the Statistics Canada Health Profile, December 2013 [17] and constructed variables to reflect the population structure of each PHU: percentage of population in each age category (based on ten-year age groups), percentage male population, and population density [15] (Additional file 1: Table S1).

#### Climate data

To capture variation in monthly temperature and precipitation over the study period, 2001–2014, we obtained estimates of mean monthly minimum and maximum temperature (^o^ C) and mean monthly precipitation (millimetres of rainfall) from Natural Resources Canada. Estimates were extracted from interpolated climate datasets for each PHU centroid (geometric centre) [18].

#### Environmental data

To assess the associations between different ecosystem types and WNV incidence, we used a 27 class raster file for land cover (year 2000) from the Ontario Ministry of Natural Resources [19]. The percentage of land in each land cover class was calculated for each PHU using the ‘Tabulate area’ tool in ArcMap 10.2 (ESRI, Redlands, USA). To reduce the risk of multicollinearity, the number of land cover class variables was reduced by combining highly correlated categories after examining Spearman rank correlation coefficients generated using SAS 9.4 © 2012 (SAS Institute Inc., Cary, NC, USA); classes with Spearman correlation values above 0.7 were aggregated. The final categories included: Swamp (coniferous swamp, deciduous swamp), Coniferous (mainly coniferous, dense coniferous forest, sparse coniferous forest), Cuts and burns (old cuts and burns, recent burns, recent cutovers), Bog and fen (open bog, treed bog, treed fen), Deciduous (sparse deciduous forest, mainly deciduous forest), Dense deciduous forest, Open fen, Settlement and developed land, and Water.

#### Mosquito surveillance data

Data on the frequency and location of mosquito trapping and results of mosquito species identification and WNV testing were obtained from the Vector Surveillance dataset maintained by Public Health Ontario. WNV surveillance in Ontario is conducted by individual PHUs during the transmission season, with mosquito traps placed at pre-determined locations for weekly mosquito collection. Collected mosquitoes are pooled together by trap-date and species, with up to 50 individual female mosquitoes per pool, and WNV vector species are tested for WNV [20]. The annual percentage of WNV-positive mosquito pools for each PHU was calculated as the total number of WNV-positive mosquito pools divided by the total number of pools tested in a given year and PHU.

#### Statistical modelling

SAS statistical software (using the procedure Glimmix) was used for analyses (SAS 9.4 © 2012, SAS Institute Inc., Cary, NC, USA). We constructed a generalized linear mixed model with Poisson distribution using annual WNV incidence per PHU as the dependent variable and the natural log of the population size as an offset. Candidate predictor variables were entered as fixed effects. To account for spatial autocorrelation, we specified PHU as a random effect with a spherical spatial correlation structure based on latitude and longitude. We accounted for correlation in repeated measures on the same PHU over time using year as a random effect with the residual statement. To reduce the number of candidate variables we first constructed models exploring each category of variables (i.e. land cover, population structure, mean monthly maximum and minimum temperature, mean monthly precipitation). We then tested significant variables in a multivariable model using a stepwise selection procedure with inclusion and exclusion significance levels set at 10%. Goodness of fit was evaluated using studentized and conditional studentized residuals. A separate model was constructed to investigate the association between ‘early season’ climate variables (i.e. those that are measurable prior to the onset of the WNV transmission season), and annual WNV incidence. In this model, we considered monthly temperature and precipitation indices from January to April that were significant in the category-specific models, and excluded estimates of WNV-positive mosquito pools.

## Results

### Data characteristics

Environmental and population structure characteristics of the study area PHUs during the study period are described in Table 1. The average cumulative incidence of WNV per PHU was 7.13 per 100,000 (7.17 SD), with the highest incidence in the Windsor-Essex County PHU (Fig. 1). Mean minimum monthly temperature ranged from − 9.90 °C (4.23 SD) in January to 15.54 °C in July (1.59 SD). Mean maximum monthly temperature ranged from − 1.99 °C (3.24 SD) in January to 27.02 °C (1.84 SD) in July. The highest mean monthly precipitation was observed in July (86.43 mm; 28.90 SD) and the lowest was 57.43 mm (24.64 SD) in February. Human WNV cases were concentrated during the transmission season (mid-April to October).

**Table 1:** Descriptive characteristics of 29 southern Ontario Public Health Units (PHUs), 2002–2013

Category	Variable	Mean	Standard Deviation
Cumulative Human WNV^a^ Incidence (cases per 100,000 population)		7.13	7.17
Land Cover (%; *N* = 29)	Swamp	2.12	2.15
	Coniferous	7.56	8.82
	Bog and fen	0.17	0.37
	Deciduous	6.18	6.87
	Dense deciduous forest	11.55	4.75
	Open fen	0.17	0.28
	Settlement and developed land	6.68	15.39
	Water	3.30	4.25
Population Structure (*N* = 29)	% Ages 1–14	16.64	1.67
	% Ages 15–24	13.0	0.83
	% Ages 25–34	11.17	1.43
	% Ages 35–44	12.49	1.43
	% Ages 45–54	16.19	0.60
	% Ages 55+	30.52	4.34
	% Male Population	49.00	0.44
Monthly Mean Minimum Temperature (^o^C^b^; *N* = 348)	January	−9.90	4.23
	February	−9.82	3.08
	March	−4.76	2.84
	April	1.47	1.46
	May	7.27	1.79
	June	13.00	1.55
	July	15.54	1.59
	August	14.51	1.47
	September	10.73	1.53
	October	4.85	1.87
	November	−0.05	1.94
	December	−5.94	2.86
Monthly Mean Maximum Temperature (^o^C; N = 348)	January	−1.99	3.24
	February	−1.02	2.19
	March	4.99	2.93
	April	12.38	1.87
	May	19.02	1.97
	June	24.19	1.56
	July	27.02	1.84
	August	25.85	1.24
	September	22.00	1.68
	October	14.03	1.81
	November	7.95	1.93
	December	0.93	2.40
Monthly Mean Precipitation (mm;^c^ *N* = 348)	January	68.33	24.33
	February	57.43	24.64
	March	60.10	24.50
	April	75.87	29.46
	May	82.95	31.27
	June	79.70	31.90
	July	86.43	28.90
	August	70.00	25.57
	September	84.52	31.31
	October	86.10	34.16
	November	80.15	29.62
	December	78.80	26.75

^a^WNV = West Nile Virus

^b^°C = degrees Celsius

^c^mm = millimetres

### Statistical modelling

Based on category-specific models, we identified that several monthly climate indices and the annual percentage of WNV-positive mosquito pools were significantly associated with annual human WNV incidence, while associations with different land cover classes and population structure variables were not significant (Table 2). August and April mean maximum temperature and March, July and August mean minimum temperature were not initially found to be significant but were tested in the final multivariable model based on their relevance to the mosquito lifecycle. In the final multivariable model, we identified that several climatic indices and the percentage of annual WNV-positive mosquito pools were significantly predictive of human WNV cases, after adjusting for spatial dependence and repeat measures (Table 2). A one-degree Celsius increase in mean minimum temperature for January, February, July and August increased the risk of human WNV infection by 5% (RR = 1.05; CI: [1.00, 1.10]), 58% (RR = 1.58, CI: [1.42, 1.75]), 20% (RR = 1.20; CI: [1.05, 1.40]) and 41% (RR = 1.41; CI: [1.02, 1.55]), respectively. Increases in March and April mean minimum temperatures by one degree Celsius decreased risk by 22% (RR = 0.78; CI: [0.71, 0.83]) and 47% (RR = 0.53; CI: [0.42, 0.63]), respectively. An increase in April mean maximum temperature by one degree Celsius was found to decrease risk by 15% (RR = 0.85; [0.75, 0.94]), while a one degree Celsius increase in August mean maximum temperature increased risk by 20% (RR = 1.02; CI: [1.02, 1.54]). The influence of precipitation was less clear, with a one-millimetre increase in February precipitation associated with a 1% (RR = 0.99; CI: [0.98, 0.99]) decreased risk. A 1 % increase in the annual percentage of WNV-positive mosquito pools increased risk by 7% (RR = 1.07, CI: [1.04, 1.11]). All category-specific and multivariable model results are summarized in Table 2. Plots of residuals revealed some overdispersion indicated by a chi-square/ degrees of freedom value of 1.58, particularly for the Windsor-Essex region.

**Table 2:** Results of the category-specific and final nultivariable Poisson regression analyses to identify predictors of annual WNV incidence in southern Ontario Public Health Units (PHUs), 2002–2013

		Category-Specific		Multivariable	
Category	Variable	Relative Risk	95% CI	Relative Risk	95% CI
Land Cover (%)	Swamp	1.13	[0.72,1.77]	–	–
	Coniferous	0.86	[0.71,1.04]	–	–
	Bog and fen	2.17	[0.12,38.69]	–	–
	Deciduous	1.09	[0.92,1.29]	–	–
	Dense deciduous forest	0.94	[0.85,1.03]	–	–
	Open fen	0.92	[0.13,6.48]	–	–
	Settlement and developed land	1	[0.98,1.03]	–	–
	Water	0.99	[0.90,1.09]	–	–
Population Structure	% Ages 1–14	0.94	[0.64,1.38]	–	–
	% Ages 15–24	0.98	[0.54,1.78]	–	–
	% Ages 25–34	0.71	[0.42,1.20]	–	–
	% Ages 35–44	1.5	[0.87,2.61]	–	–
	% Ages 45–54	0.45	[0.20,1.02]	–	–
	% Ages 55+	0.97	[0.90,1.04]	–	–
	% Male population	0.53	[0.19,1.47]	–	–
Monthly Mean Minimum Temperature (°C)	January	1.20	[0.89,1.60]	1.05	[1.00,1.10]
	February	1.82	[1.31,2.53]	1.58	[1.42,1.75]
	March	0.82	[0.56,1.21]	0.78	[0.71,0.83]
	April	0.50	[0.31,0.79]	0.53	[0.42,0.63]
	May	1.03	[0.74,1.42]	–	–
	June	1.11	[0.61,2.03]	–	–
	July	0.98	[0.64,1.51]	1.20	[1.05,1.40]
	August	1.46	[0.91,2.34]	1.41	[1.02,1.55]
	September	0.96	[0.58,1.58]	–	–
	October	1.71	[1.05,2.77]	–	–
	November	0.64	[0.35,1.16]	–	–
	December	0.70	[0.42,1.15]	–	–
Monthly Mean Maximum Temperature (°C)	January	0.86	[0.59,1.27]	–	–
	February	0.91	[0.67,1.25]	–	–
	March	0.87	[0.64,1.19]	–	–
	April	1.08	[0.82,1.43]	0.85	[0.75,0.94]
	May	1.26	[0.94,1.68]	–	–
	June	0.60	[0.37,0.98]	–	–
	July	1.38	[0.87,2.20]	–	–
	August	1.10	[0.67,1.80]	1.2	[1.02,1.54]
	September	1.60	[1.06,2.44]	–	–
	October	0.79	[0.53,1.17]	–	–
	November	1.04	[0.66,1.65]	–	–
	December	1.50	[0.89,2.51]	–	–
Monthly Mean Precipitation (mm)	January	0.99	[0.98,1]	–	–
	February	0.98	[0.97,1]	0.99	[0.98,0.99]
	March	0.99	[0.98,1]	1	[0.97,1.01]
	April	0.99	[0.99,1]	–	–
	May	1.00	[1,1.02]	–	–
	June	0.99	[0.98,1]	–	–
	July	1.00	[0.99,1]	–	–
	August	1.00	[1,1.01]	–	–
	September	1.01	[1,1.02]	–	–
	October	1.00	[1,1.01]	–	–
	November	1.01	[1,1.02]	–	–
	December	1.00	[0.99,1.01]	–	–
Annual Percent Positive Mosquito Pools		1.29	[1.27,1.34]	1.07	[1.04,1.11]

Results of the ‘early season’ model using only variables available prior to the WNV season (i.e. those measures that would be available to public health practitioners before the onset of WNV transmission in a given year – which effectively excludes mosquito surveillance data) are presented in Table 3. This model showed similar results to the full model; however, the relative risks were noticeably different. Notably, an increase in mean minimum temperature by one degree Celsius in February resulted in an 91% (RR = 1.91; CI:[1.73, 2.12]) increase in WNV incidence for any given PHU. An increase in early spring (March and April) mean minimum temperatures by one degree Celsius decreased risk of WNV by 35% (RR = 0.65; CI:[0.60, 0.70]) and 52% (RR = 0.48; CI: [0.39, 0.60]), respectively. Finally, we found that increasing the February mean precipitation by 1 mm decreased WNV risk by 3% (RR = 0.97; CI:[0.97, 0.98]). No mean monthly maximum temperature values or precipitation values other than February were found to be significantly associated with WNV incidence.

**Table 3:** Significant predictors of annual WNV Incidence in southern Ontario Public Health Units (PHUs), 2002–2013, based on Poisson modelling of pre-season climate indices

Category	Variable	Relative Risk	Confidence Interval
Monthly Mean Minimum Temperature (°C)	January	1.08	[1.03, 1.12]
	February	1.91	[1.73, 2.12]
	March	0.65	[0.60, 0.70]
	April	0.48	[0.39, 0.60]
Monthly Mean Precipitation (mm)	February	0.97	[0.97,0.98]

## Discussion

To address the knowledge gap on population level predictors of human WNV incidence in Ontario, we applied a multivariable mixed modelling approach that incorporated key environmental and population factors at the PHU level. Monthly climate indices, particularly February, March and April minimum temperature and February precipitation, and the annual percentage of WNV-positive mosquito pools in a given PHU, were significantly predictive of human WNV incidence across PHUs of the southern portion of Ontario. When considering only ‘early season’ variables, which effectively excludes mosquito surveillance data, January and February mean minimum temperatures were of primary importance, highlighting the utility of climate data in predicting WNV risk in the upcoming transmission season.

Our findings are similar to previous studies, which also found higher winter temperatures to be significantly predictive of increased rates of WNV transmission in an upcoming season. For example, Wimberly et al. found that winter temperature variables had the greatest influence on West Nile virus human infection rate in the United States in 2014; December and January temperatures in particular were most significant [21]. Similarly, Manore et al., found mean minimum January temperature to be a highly significant predictor of human WNV [9]. Our analysis indicates that January and February temperatures may be relevant predictors for WNV incidence in the southern portion of Ontario. Temperatures during the winter months have a considerable impact on the ability of WNV to survive into the spring, and in colder years effective overwintering of mosquitoes is lessened [22].

Maximum temperatures also had a significant influence on WNV activity. It was found that lower April and higher August mean maximum temperatures were significantly related to human WNV incidence. The significance of the April mean maximum temperature may be related to virus amplification in the avian host, which is believed to occur in the early spring [8]. Warmer temperatures during this period may be unfavourable since spring temperatures that are too warm might result in faster melting of snow which could dilute nutrients in standing water or flush *Culex* breeding sites, impeding larval proliferation [23]. The causal association between warmer August maximum temperatures and human WNV infection is expected, as warmer summer temperatures increase mosquito abundance and biting rates and decrease viral amplification time [24].

In addition to temperature associations, precipitation was found to contribute significantly to increased human WNV risk, albeit more subtly. We found that lower February and March mean precipitation was associated with higher WNV incidence in a given year. While this is in contrast to some studies in the United States that found increased March precipitation to be associated with outbreaks, these results may be attributable to the variable influence of precipitation across the study region and differences in primary vector species [21]. Further to this, it has been proposed that early spring drought may concentrate vectors and hosts around pools of water, and allow for low populations of vector predators [25].

Finally, we found that the annual percentage of WNV-positive mosquito pools was significantly predictive of human WNV incidence, confirming our expectation that higher mosquito infection rates should result in more transmission events. This has been previously remarked by Brownstein et al.*,* who noted that mosquito surveillance data is the most sensitive marker for human risk with positive mosquito pools accounting for 38% of human risk [26], and suggested that this data should be included in any surveillance system. The association between WNV-positive mosquito pools and human WNV case counts has also been noted by Rochlin et al., who found a strong association between human risk and proximity to a single WNV-positive mosquito pool [10], and Liu et al., who found that presence of a WNV-positive pool in the last 30 days was significantly predictive of risk for human infection [14]. The results of our study indicate that a 1% increase in annual WNV-positive mosquito pools would result in a 7% increase in the annual human WNV incidence rate.

This study identifies several predictors of human WNV incidence in southern Ontario that can be of practical use for public health. Importantly, readily available data on climate indices may be used by public health officials for predicting more severe WNV seasons at the PHU level. Limitations include the fact that data were aggregated at the PHU level, which may have masked smaller-scale variations in WNV incidence and predictor variables (including land cover and population structure) and hence associations. Avian data was not included in the analyses due to paucity of data. Since birds are the main reservoir hosts of WNV, bird dynamics may substantially influence seasonal risk, and previous studies have found links between bird community composition and WNV incidence [27]. Future studies may seek to include this type of data to produce a more comprehensive model. In addition, PHU-level census data availability was restricted to 2011, which may have limited the importance of population structure variables in our analysis. Finally, while the proposed multivariable model was successful in identifying key predictors of human WNV incidence in southern Ontario PHUs, some overdispersion was noted particularly for the most southern PHUs, which could affect the reliability of model estimates for these areas.

Despite these limitations, the variables identified as significant in our model may be useful for public health planning. In practical terms, this could entail monitoring of monthly average temperature and precipitation trends in an area, and comparing the data to normal or historical values. Identification of higher than average minimum winter temperatures and lower than average spring precipitation at the PHU level could serve as indicators of elevated risk of WNV in an upcoming transmission season. These early year estimates of virus activity could then be used to inform decisions regarding the quantity of resources that could be put towards in-season risk measures based on mosquito surveillance. This is important, since small scale weather events such as sudden heavy rains or short cold periods, which affect mosquito survival, can have a large impact on WNV risk [8]. While early season predictors may be useful, our results support the ongoing monitoring of climate and entomological indices to more accurately predict WNV risk at the local scale.

## Conclusion

Overall, although WNV does not consistently pose a public health risk to the majority of Ontarians, our results indicate that by using measures of risk that are detectable early in the year it may be possible to estimate the level of WNV activity for the upcoming season, allowing PHUs to tailor appropriate preventive strategies and decrease risk to public health.

## Additional file

Additional file 1: **Table S1.** Sex and Age Distribution by Public Health Units (PHUs) in Southern Ontario, 2002–2013. (DOCX 17 kb)
