# Supplementary material for: Factors associated with human West Nile virus infection in Ontario: a generalized linear mixed modelling approach
Source: BMC Infect Dis. 2018 Mar 27;18:141. doi: 10.1186/s12879-018-3052-6 (PMC5872497; doi:10.1186/s12879-018-3052-6)
Supplement: Supplementary file 1 — Table S1. Sex and Age Distribution by Public Health Units (PHUs) in Southern Ontario, 2002–2013. (DOCX 17 kb) [file 12879_2018_3052_MOESM1_ESM.docx]

Additional Table 1. Sex and Age Distribution by Public Health Units (PHUs) in Southern Ontario, 2002-2013

| **Health unit** | **Ages 1-14 (%)** | **Ages 15-24 (%)** | **Ages 25-34 (%)** | **Ages 35-44 (%)** | **Ages 45-54 (%)** | **Ages 55+ (%)** | **Male population (%)** |
| --- | --- | --- | --- | --- | --- | --- | --- |
| Brant County | 18.18 | 13.15 | 11.73 | 12.82 | 15.59 | 28.54 | 48.59 |
| Chatham-Kent | 17.02 | 12.52 | 10.40 | 11.53 | 16.25 | 32.28 | 48.63 |
| Durham | 18.59 | 14.28 | 11.61 | 14.09 | 17.34 | 24.10 | 48.73 |
| Elgin-St. Thomas | 19.10 | 12.76 | 10.71 | 12.91 | 15.54 | 28.98 | 49.18 |
| Eastern Ontario | 16.15 | 12.63 | 9.91 | 12.38 | 17.52 | 31.41 | 49.17 |
| Grey Bruce | 15.06 | 12.01 | 9.63 | 10.25 | 15.87 | 37.18 | 49.27 |
| Halton | 19.62 | 12.56 | 11.36 | 15.78 | 16.10 | 24.57 | 48.58 |
| Hamilton | 16.49 | 13.96 | 12.33 | 12.88 | 15.92 | 28.43 | 48.81 |
| Halimand-Norfolk | 16.10 | 12.93 | 9.56 | 11.53 | 16.64 | 33.25 | 49.76 |
| Haliburton, Kawartha, Pine Ridge District Health Unit | 13.64 | 11.34 | 8.46 | 10.34 | 16.75 | 39.47 | 49.19 |
| Hastings Prince Edward County | 15.14 | 12.04 | 10.10 | 11.27 | 16.51 | 34.94 | 48.78 |
| Huron | 17.06 | 12.61 | 9.79 | 10.41 | 15.06 | 35.07 | 49.28 |
| Kingston, Frontenac and Lennox and Addington | 14.96 | 13.92 | 12.18 | 12.21 | 15.78 | 30.96 | 49.09 |
| Lambton | 15.59 | 12.93 | 10.55 | 11.03 | 15.78 | 34.12 | 48.70 |
| Leeds, Grenville and Lanark District Health Unit | 15.15 | 11.67 | 8.92 | 11.99 | 16.98 | 35.29 | 48.76 |
| Middle-sex London | 16.58 | 14.43 | 13.11 | 12.78 | 15.64 | 27.47 | 48.47 |
| Niagara | 15.48 | 12.92 | 10.58 | 12.21 | 15.84 | 32.97 | 48.39 |
| Ottawa | 16.82 | 14.07 | 13.47 | 14.11 | 16.10 | 25.44 | 48.50 |
| Oxford | 18.25 | 12.70 | 11.38 | 12.52 | 15.73 | 29.41 | 49.25 |
| Perth District | 18.29 | 13.33 | 11.16 | 11.89 | 15.55 | 29.78 | 49.00 |
| Peel | 19.66 | 14.42 | 13.17 | 15.13 | 15.98 | 21.64 | 49.13 |
| Peterborough County-City | 14.27 | 13.42 | 10.66 | 10.76 | 15.49 | 35.41 | 48.16 |
| Simcoe Muskoka District | 16.71 | 12.96 | 10.72 | 13.04 | 16.79 | 29.77 | 49.15 |
| Toronto | 15.33 | 12.75 | 15.79 | 14.83 | 15.25 | 26.04 | 48.01 |
| Waterloo | 18.33 | 14.16 | 13.81 | 14.25 | 15.38 | 24.06 | 49.26 |
| Wellington-Dufferin-Guelph | 18.40 | 14.00 | 11.73 | 13.57 | 16.28 | 26.07 | 49.10 |
| Windsor-Essex county | 17.62 | 13.41 | 11.33 | 13.72 | 15.81 | 28.12 | 49.19 |
| York | 18.61 | 13.92 | 11.52 | 15.29 | 16.83 | 23.84 | 48.88 |
